# Recent advances in research in the rumen bloat of ruminant animals fed high-concentrate diets

**DOI:** 10.3389/fvets.2023.1142965

**Published:** 2023-03-23

**Authors:** Yusu Wang, Lizhi Wang, Zhisheng Wang, Bai Xue, Quanhui Peng, Rui Hu, Tianhai Yan

**Affiliations:** ^1^Institute of Animal Nutrition, Sichuan Agricultural University, Chengdu, Sichuan, China; ^2^Ruminant Nutrition and Feed Analysis Laboratory, Agri-Food and Biosciences Institute, Hillsborough, United Kingdom

**Keywords:** rumen bloat, high concentrate diet, foam, microorganism, tannin

## Abstract

Rumen bloat is the most common digestive disorder in fattening ruminants, which is responsible for around 2–3 % of deaths in the ruminants industry and is therefore considered to be a serious threat to ruminant farming. The root cause of rumen bloat caused by feeding high concentrate dies would be attributed to the production of a large amount of stable foam during the fattening period. The exact mechanism of rumen foam formation has yet to be investigated. Proteins, polysaccharides and carboxylates derived from feed, and synthesized by microbes during the rumen fermentation may act as foaming agents or stabilizers in the formation progress of rumen foam. Supplementation of condensed tannins and other additives can be an effective way to prevent feedlot bloat induced by feeding high concentrate diets.

## Introduction

In recent years, the concentrate input in ruminant production has been increasing for various reasons such as improving animal performance and meat quality. The long-term feeding of high concentrate diets (HCDs) can induce a series of gastrointestinal diseases in ruminant animals, among which rumen bloat is a very common problem. Under normal circumstances, although rumen fermentation produces large amounts of gases, although rumen fermentation produces large amounts of gases, ruminants can expel them out of their bodies through eructation (1), so gases do not accumulate in large quantities in the rumen. However, sometimes when the gas emission in the rumen is restricted with the production rate exceeding the emission rate, gases can accumulate in the rumen. If this situation persists, it can lead to severe distention of the rumen, then ruminal contractions are inhibited and result in ruminal atony. When the pressure in the rumen reaches up to 70 mm Hg (2), the animal can suffer from rumen bloat. An inflated rumen can mechanically interfere with respiration, which may be caused by excessive absorption of carbon dioxide into body from the rumen, leading to respiratory distress and possibly eventual death. According to pathogenesis, rumen bloat can be classified into primary and secondary bloat. The bloat caused by feeding HCDs belongs to primary bloat and is also known as feedlot bloat. This article aims to present a review of the current knowledge about pathogenesis and prevention of feedlot bloat.

## High concentrate diets and rumen bloat

The use of HCDs is becoming increasingly common in the modern cattle and sheep farming. For example, in order to produce snowflake beef, the proportion of concentrates in the diet of cattle in the late fattening period has been increased to over 90%. Long-term feeding of high-concentrate diets can significantly increase rumen bloat in ruminants. The incidence of this kind of nutritional metabolic disease, although high, is insidious as the vast majority is subacute and difficult to be detected by the senses alone (3). Under high-concentrate feeding conditions, cattle and sheep are likely to be in subacute rumen bloat state in most of time, with clinical manifestations only following further episodes. Because of this, although the mortality caused by rumen bloating was only 0.1% (4), a study showed that it caused an annual economic loss of 180 million US dollars in Australia and 310 million US dollars in the United States (5), mainly due to the significant decline in growth performance during onset and treatment. Therefore, farmers' concern to feedlot bloat leads them to limit the supply of cereal grains to fattening animals. Although this can effectively prevent the occurrence of rumen bloat, it reduces the performance of animals, and the economic loss caused by this may even exceed the loss caused by rumen bloat (6). Another reason for the low incidence of clinical rumen bloat is the widespread application of antibiotics in feed such as Monensin. This is because monensin effectively inhibits the growth of lactic acid bacteria, making lactic acid less able to reduce rumen contraction and peristalsis, and therefore monensin are significantly effective in suppressing rumen bloat (7–9). However, a total ban on the application of antibiotics in feed seems unpreventable, and many countries around the world, such as China, have already implemented it. Therefore, it is particularly urgent to understand the mechanism of feedlot bloat and then develop feeding technologies to prevent it instead of use of antibiotics.

## Pathogenesis of feedlot bloat

According to the current scientific knowledge and production experience, there are two opinions on the cause of rumen bloat induced by feeding high-concentrate diets: one attributed feedlot bloat to too much and too fast gas production in the rumen, and other attributed it to the formation of large number of stable foams in the rumen.

### Ruminal gas production and feedlot bloat

Ruminal bloat is the result of excessive gas accumulation in the rumen. Gases produced in the rumen are normal by-products of microbial fermentation and consist mainly of carbon dioxide (76%), methane (22%) and nitrogen (2%). The normal rumen gas production rate is typically 0.2–2.0 L/min (7), and they can be excreted from the rumen under normal conditions by belching. To date no *in vivo*' trials have been performed to study the correlation between the production of rumen gas, gas production rate and feedlot bloat. It is generally accepted that HCDs contains high starch content, which is likely to produce more gas in the rumen than cellulose in an equal mass. As the proportion of concentrate in the diet increases, the metabolizable (net) energy concentration of the diet also increases and the amount of food ingested by the animal is bound to decrease. On the other hand, methane gas emissions per kg of dry matter intake are significantly reduced with an increase in the proportion of concentrate in the die (10). Therefore, feeding a high concentrate diet with a lower intake and lower methane emissions per kg of dry matter intake may not necessarily result in an increase in absolute daily rumen gas production in ruminants. Even if it does increase, its magnitude will not be significant. The only thing that is certain that rate of rumen gas production on HCDs is faster than on coarse diets. However, one study found that although the rate of digestion and gas production in the rumen was faster in stream-flaked barley than in whole barley, the incidence of rumen bloat was significantly lower in stream-flaked barley diets than in whole barley diets (11). Ruminal fermentation rates and degree were greater in wheat than in barley, sorghum or maize (12). However, no trials to date have found significant differences in inducing rumen bloat by dietary grain types. The inhibitory effect of tannin on rumen gas production was significantly higher than that of monensin, but the inhibitory effect of monensin on rumen bloat was significantly better than that of tannin (13). These results indicate that feeding high-concentrate diet does not necessarily lead to a significant increase in daily gas production in the rumen, and high or fast rumen gas production does not necessarily lead to rumen bloat.

### Rumen foam and feedlot bloat

**Figure 1:**
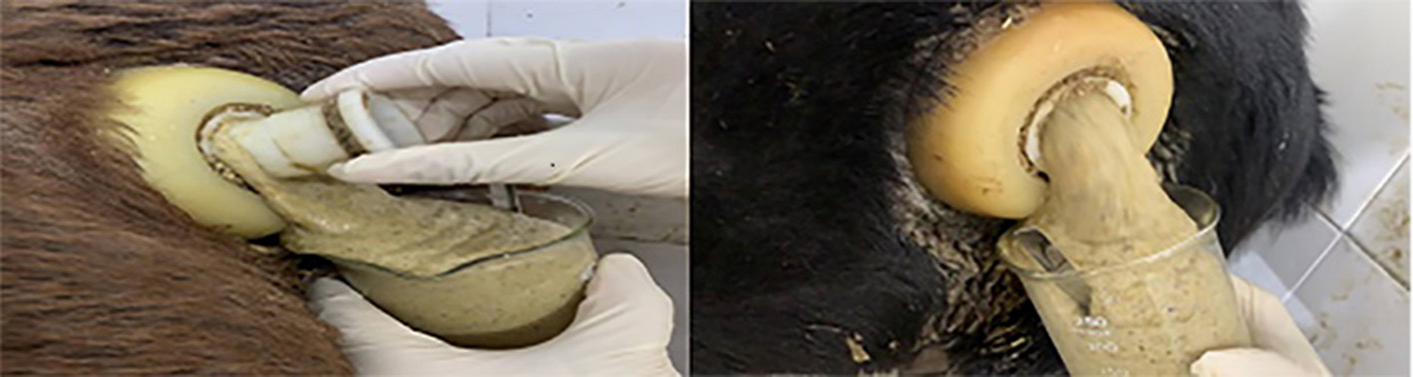
Ruminal fluid coming from ruminal animals with feedlot bloat (14).

**Figure 2:**
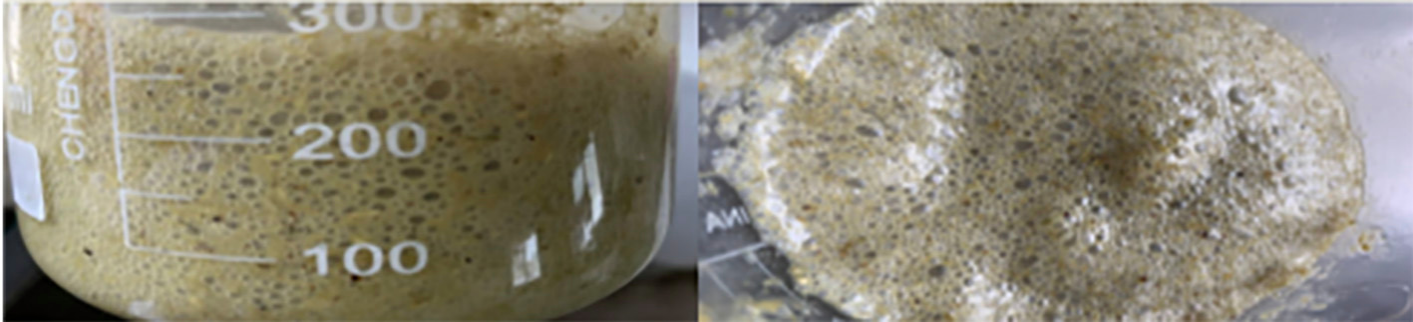
Rumen bloat in goats with fistulas fed high concentrate diets (14).

Foam is a gas trap, and the formation of large amounts of stable foam can reduce the normal flow and emission rate of rumen gases. During the progress of foam formation, the gas is trapped in the rumen content and forms a small bubble-like emulsion of approximately 1–2 mm in diameter (Figure 1). With high concentrate feeding, the foam can expand until to filling the entire rumen, thus inhibiting the excitability of the nerve endings that control the opening of the esophagus, consequently resulting in an obstruction of normal belching behavior (15), which, in return, further inhibits the release of fermentation gas from the rumen. When the accumulation of fermentation gas reaches a certain extent, the rumen distends and is stretched, this produces rumen bloat. In the trials with rumen fistulated goats, Xu, (16) found that goats fed a HCD that resulted in rumen bloat had a rumen full of foam. When the lid of the rumen fistula was opened, a large amount of foam-laced rumen contents gushed out (Figure 2). The use of a rumen fistula provides very visual evidence of the presence of large amounts of foam in the rumen. The concept that rumen bloat caused by feeding HCDs is a foam type bloat has gradually gained acceptance (17), but the reasons for foam formation have not yet been deciphered. To form a large number of stable foam in the rumen, a foaming agent and a stabilizer are required which act, respectively, to promote the production of foam from the rumen fluid, and to keep the foam stable and unbroken. Since the rumen of ruminants does not have secretory function, substances in rumen can only come from saliva, feed, degradation products of feed inrumen and *de novo* synthesis of rumen microorganisms. The previous research has proved that saliva of ruminants has a weak inhibitory effect on foam production (18), so foaming agents and foam stabilizers can only come from the other three sources.

Although there are many types of foaming agents and foam stabilizers (19), most of them do not exist in natural feed, cannot be synthesized by rumen microorganisms, and cannot be produced by fermenting feed. Based on the knowledge of physical chemistry, feed science and animal physiology, it is recognized that the three main types of substances that can act as foam agents or foam stabilizers in the rumen are proteins, carboxylates, and soluble polysaccharides (16).

#### Protein

The protein itself is a low activity surfactant and its peptide chains, when stretched on the liquid surface, will form a two-dimensional protective network through the interaction of intra- and inter-molecular forces, which can maintain the stability of the foam (19, 20). Recent studies found that when goats were fed high-concentrate diets, the protein content of rumen foam was much higher than that of raw rumen fluid, and the foaming performance of the rumen fluid was significantly positively correlated with the concentration of protein in the rumen fluid (21). These results suggest that protein can be enriched on rumen fluid foam and protein content of rumen fluid is an important factor influencing rumen fluid foaming performance. There are three sources of protein in rumen fluid: saliva, feed, and *de novo* synthesis by rumen microorganisms. Since proteins in ruminant saliva have anti-foam effect, proteins that promote rumen foam production are mainly derived from diet and microorganisms. The proteins of microbial origin in rumen include microbial body protein and microbial secreted protein. Almost all rumen microorganisms can synthesize their own body proteins, but so far there is no evidence indicates that rumen foam formation is related to microbial body protein because they can hardly be dissolved in the rumen fluid. Many ruminal microorganisms can synthesize secretory proteins (22), which are highly soluble. When feeding ruminants with HCDs, changes in the structural composition of rumen microorganisms may lead to an increase of secretory proteins and the concentration of protein in rumen fluid, which promotes the production of rumen foam. As the true protein in ruminant saliva is almost negligible, the only possible sources of protein in the rumen are from diets and microorganisms.

Although rumen microorganisms can synthesize a large amount of mycoprotein every day, there has been, so far, no evidence indicating that rumen foam formation is related to mycoprotein. Ruminal microorganisms can also synthesize large amounts of secretory proteins. If the formation of rumen foam is related to the secreted proteins of microorganisms, and only high-concentrate diet can cause the formation of large amounts of foam in the rumen, this implies that high-concentrate diets may induce rumen foam by changing the structure and composition of rumen microorganisms, affecting the types or amounts of proteins secreted, and ultimately inducing rumen foam. Whether this conjecture is correct remains to be proven.

Feed ingredients of ruminants, whether roughage or concentrate, all contain some amount of protein. If proteins are required to maintain foam stability, they must first be sufficiently dissolved, as the stability of the liquid film can only be maintained if the foam level reaches a sufficient concentration. Moreover, proteins should be hydrophobic, pliable and disordered so that they can easily concentrate at the gas/liquid interface and form a film with a certain degree of elasticity (23). Since the majority of proteins in ruminant feed materials, such as gluten and glycolin in corn, wheat and rice, and globulin in soybean and cottonseed, are almost insoluble in water (24) and have ordered structures, the proteins in natural feed materials have poor foaming properties. However, the fermentation of proteins in feeds by rumen microorganisms increases the hydrophobicity of protein molecules, which may improve the foam ability of protein. At the same time hydrolysis may also increase the cross-linking of the polypeptide chains and the viscosity between the lamellae, improving the stability of the foam. The formation of beer foam and rumen foam has a similar principle. Studies have shown that the main component of beer foam is protein, of which 40% is lipid transfer protein 1 (LTP1). The LTP1 is a key substance in maintaining the stability of beer foam (25), but the structure of LTP1 isolated from beer is completely different from that of LTP1 in the natural state of barley. During microbial fermentation of barley, LTP1 protein undergoes defolding and denaturation, increasing its hydrophobicity, amphiphilic character and solubility, thus transforming LTP1 into a form more conducive to improved foam stability (26). The role of proteins in promoting rumen foam production and maintaining foam stability needs further study.

#### Carboxylates

Carboxylates, commonly known as soap, is an anionic surfactant with excellent foaming properties. Carboxylates do not exist in natural feedstuff or rumen microorganisms. However, the germs of maize and wheat contain a large amount of fatty acids and the microbial breakdown of carbohydrates in the rumen also produces a large amount of fatty acids. Ruminal acidosis can be induced by feeding HCDs. In order to raise the pH of the rumen fluid, animal producers often add high doses of sodium bicarbonate to HCDs. Sodium bicarbonate and other mineral salts in the feed react with fatty acids to form Carboxylates (27), which reduces the risk of rumen acidosis but may thereby provide an excellent foaming agent in the rumen. For example, oleic acid has a hydrophilic-lipophilic balance (HLB) value of only 1, which is an anti-foaming agent at its alone. However, when reacting with sodium bicarbonate to produce sodium oleate, the product has an HLB value of 18 (27), turning it into a high-performance foaming agent. Thus, we may inadvertently create the conditions for feedlot bloat while preventing rumen acidosis in HCDs. However, whether this is in fact the case, and what exactly the surface active substances are those cause the reduced surface tension of the rumen fluid in HCDs, requires further research.

#### Soluble polysaccharides

Soluble polysaccharides dissolve in water resulting in a significant increase in the viscosity of the solution (28). The viscosity of the solution affects the flow rate of the foam film, the higher the viscosity of the solution, the slower the rate of drainage flow from the surface of the foam produced and the more stable the foam (29). In addition, the higher the viscosity of the foam film, the more airtight and the more stable the foam generated will be (30). Therefore, the higher the liquid viscosity of the system, the longer life of the foam formed. Mishra et al. (31) suggested that a mucus of bacterial origin was the main substance responsible for the increased viscosity of rumen fluid. They used transmission electron microscopy to observe *in vitro* cultures of rumen bacteria and found that many of them could form a thick amorphous mucus capsule around the bacteria, which, when incubated for long periods of time, formed a distinct mucus “collar” on the walls of the rumen microbial culture tubes. This bacterial mucus was degraded by glucanase, and the addition of glucanase to the rumen fluid of cattle on HCDs resulted in an extremely significant reduction in viscosity. They therefore classified this mucus as a mucopolysaccharide. However, the exact structure of these microbial mucopolysaccharides is unknown and their role in rumen foam formation remains to be determined. Feed ingredients commonly used by ruminants contain a certain amount of soluble polysaccharides, such as β-glucan and arabinoxylan. In particular, the soluble polysaccharide content of meal and cake feedstuff is up to about 8%. It is uncertain whether these dietary sources of soluble polysaccharides play a role in maintaining the stability of the rumen foam, as their chemical properties can be maintained only when they escape degradation by rumen microorganisms, while easily soluble substances in the diet are generally preferentially broken down by microorganisms. Whether and how soluble polysaccharides in feedstuff can be prevented from being degraded by microorganisms remains to be investigated.

### Microorganism and feedlot bloat

As mentioned above, the rumen foam agent and foam stabilizer can only originate from feeds and rumen microorganisms. Numerous studies have found that rumen bloat occurs only in some animals, even when fed the same feed (32, 33). The reason for this phenomenon may be related to individual differences in the structural composition of rumen microbes, suggesting that rumen microbes play an important role in the development of rumen bloat. Although these issues have been recognized and studied previously, there is little information available on the relevance of rumen microbial structure and composition to rumen bloat due to the lack of research techniques for isolation and culture of rumen microbes. The limited information suggested a significant increase in the number of *Streptococcus bovis* in the rumen of frothy bloat animals (26), but this increase in bacteria is not necessary or a prerequisite for frothy bloat to occur (34). When ruminants consume large amounts of easily fermentable carbohydrates, the *Streptococcus bovis* population grows rapidly, allowing organic acids and enterotoxins to be produced and absorbed, leading to the acidosis. Therefore, *Streptococcus bovis* is commonly associated with acidosis, and the presence of large numbers of this bacterium does not necessarily indicate the occurrence of feedlot bloat, but may well reflect the co-occurrence of two digestive disorders, bloat and acidosis. There is little difference in rumen protozoa between high concentrate feeding cattle whether or not suffering from rumen bloat. Protozoa may not be involved in the formation of feedlot bloat because they can swallow bacteria and starch particles, thereby reducing the production of microbial mucopolysaccharide. Pitta et al. (34) compared differences in rumen microorganisms between rumen bloated and non-rumen bloated beef cattle grazed on winter wheat pastures, and found that the relative abundance of archaea, as well as *Clostridium, Eubacterium*, and *Butyrivibrio* in Firmicutes was significantly higher, and the relative abundance of *Prevotella* in Bacteroidetes was significantly lower in cattle developed rumen bloat. The symbiotic relationship between bacteria and archaea was weaker in the rumen of bloated cattle than non-rumen bloated cattle. A recent study found that the rumen fluid had significantly higher foaming performance and viscosity, significantly lower pH, and significantly different structure and composition of rumen bacteria and eukaryotes in severely bloated goats when compared to non-bloated ones under high concentrate dietary feeding conditions. At the species level, in the rumen of severely bloated goats, the relative abundance of some bacteria, such as *Bacteroides fragilis, Fibrobacter succinogenes*, and *Prevotella oralis* was significantly decreased, and some bacteria such as *Dialister invisus CAG:218* was significantly increased. The relative abundance of some eukaryotes such as *Rhizophagus irregularis* decreased significantly, while some eukaryotes such as *Aspergillus calidoustus* and *Rozella allomycis* increased significantly (21). There are still many gaps in knowledge relating to how rumen microorganisms playing a role in rumen bloat caused by feeding HCDs.

**Figure 3:**
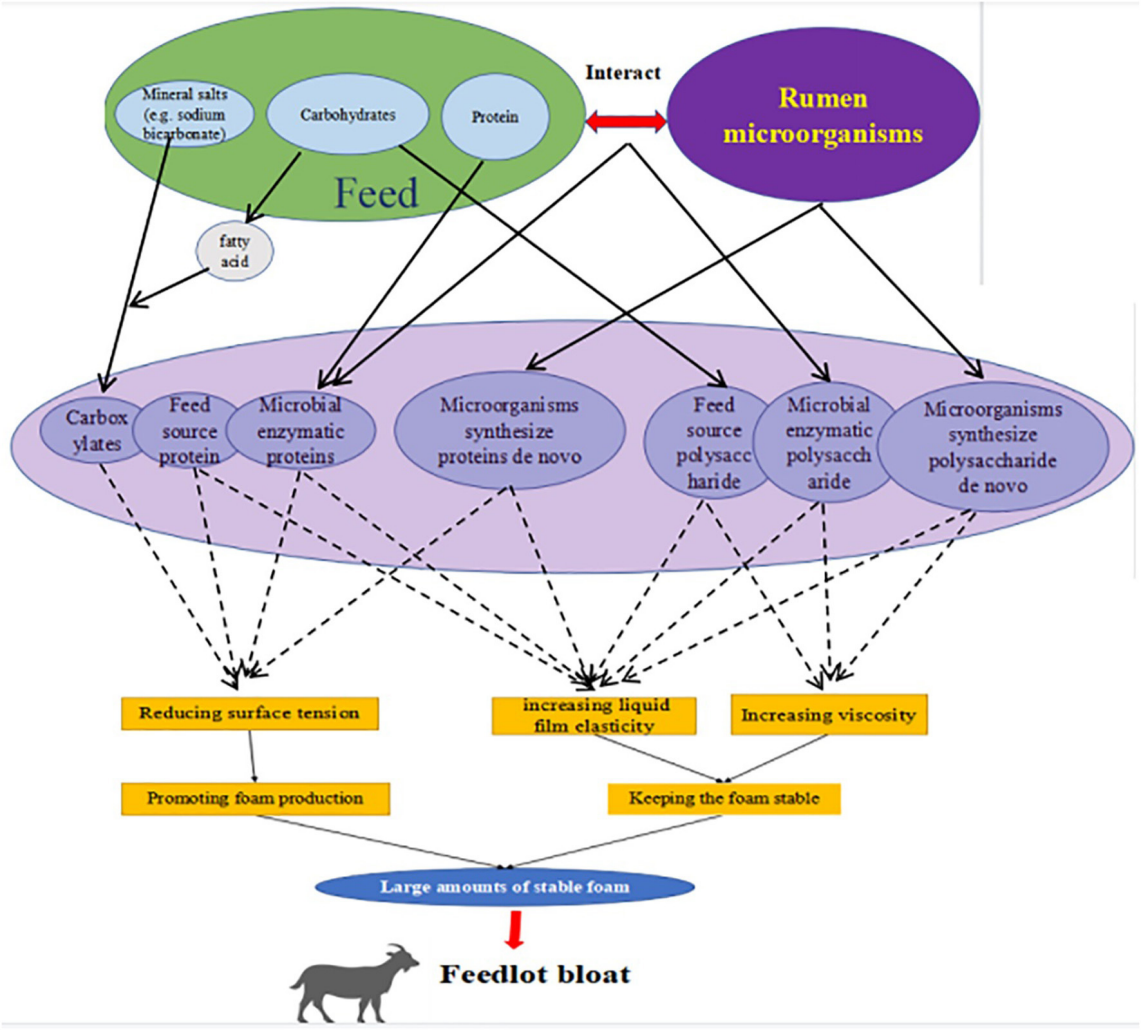
Speculative diagram of the mechanism for high-concentrate type bloat.

In summary, we speculate that the formation of large amounts of stable foam in the rumen is the most fundamental cause of rumen bloat induced by HCDs, but the mechanism of rumen foam formation is not known. We infer (Figure 3) that under HCDs, rumen digesta and rumen microorganisms, either individually or through interactions, cause changes in chemical composition of compounds dissolved in the rumen fluid (e.g., proteins, polysaccharides and carboxylates), which affect the surface tension, foaming power, viscosity and other foaming characteristics of the rumen fluid and ultimately induce the formation of large amounts of stable foam in the rumen. However, the exact type, chemical structure, source and production pathway of foaming agents and foam stabilizers in the rumen are unknown, and further research is needed to determine what role rumen microorganisms play in this process and how they do so.

## Prevention of feedlot bloat

The direct and effective method to inhibit frothy bloat is the use of antifoam agents to break down the foam in the rumen. Products such as Bloat Guard, in which the active ingredient (Poloxalene) can degrade the foam in the rumen, had been used to completely eliminate pasture bloat of grazing animals (35, 36). Alfasure, a water soluble product produced in Canada, is also effective in preventing rumen bloat. Some studies have shown that intraruminal injections of the detergents Alfasure or Anti Gaz Emulsion were very effective in the treatment of grazing type rumen bloat (37). Unfortunately, the effect of these products in the prevention of feedlot bloat is not satisfactory. A recent study found that the dietary supplementation of 0.1% Dimethy1 Silicone Oil (DSO) could significantly reduce the foam strength and foam production of rumen fluid, and did not negatively affect the rumen fermentation and the apparent digestibility of nutrients in goats (14). When DSO was added to a foaming liquid, the surface tension of this liquid (such as rumen liquid) was significantly reduced, and the stability of the foam produced by this liquid was decreased, which made the adjacent foam constantly integrated. The combination of bubbles expanded the volume of the foam until it collapsed, thereby achieving the purpose of defoaming (2). However, further research is needed to determine the appropriate dose of DSO in the diet to prevent high-concentrate type bloat.

According to the pathogenesis of high-concentrate type bloat, reducing the content of soluble protein in the rumen has the potential to prevent rumen bloat. Studies have shown that tannins can form insoluble complexes with proteins (38), which can significantly reduce the concentration of proteins in the rumen fluid. Tannins have long been considered an anti-nutritional factor due to their poor palatability and tendency to bind to proteins, sugars and metal ions to form complexes that are difficult to digest and absorb, thus reducing the digestibility of nutrients. However, studies in recent years have found that when tannins are added to the diet and their levels are controlled within appropriate limits, feed intake is not adversely affected and production performance is improved (39, 40), and the incidence of rumen bloat is significantly suppressed (41). It was found that tannins in the rumen form a complex with leaf proteins in the plant, thus effectively preventing grazing-type rumen bloat (42). In countries such as New Zealand, mixing a proportion of high tannin content Sainfoin and Lotus corniculatus with traditional alfalfa pasture significantly reduced the incidence of rumen bloat while significantly improving the production and reproductive performance of grazing sheep and deer (43). When tannins are added to the diets of captive ruminants, they form a tannin-protein complex with protein in the rumen that resists degradation by rumen microorganisms (44), which is effective in preventing feedlot bloat without reducing the digestibility of the protein in the whole gut. Because this tannin-protein complex enters the abomasum, it breaks down under acidic conditions to re-release the protein (45, 46). A recent study using a dynamic *in vitro* system combined with a digestomic approach using liquid chromatography-tandem mass spectrometry found that RuBisCo proteins from plants were protected from excessive degradation in the rumen by effective binding of tannins. The affinity of proteins to tannins depends on the characteristics of the tannin and the size of the protein, with peptides with fewer than six residues interacting less weakly with tannins, for example, chloroplast stroma proteins are more readily bound by tannins and, conversely, thylakoid membrane proteins are less accessible to tannins (47). The inhibition of rumen bloat by tannins may also be related to their ability to modulate the structural composition of rumen microorganisms and reduce the production of viscous material and rumen gases (48).

*In vitro* alfalfa saponin foam antifoaming experiments found that bovine saliva is effective in inhibiting foam formation (49). The greater the amount of saliva secreted, the less the incidence of rumen bloat. Many previous studies have proved that the content of physically available neutral detergent fiber (peNDF) in diet is significantly and positively correlated with the secretion of saliva (50, 51). peNDF is defined as neutral detergent fiber that is effective in stimulating rumen rumination and salivary secretion, and is influenced by the fiber content of the diets and more by the physical size of the fiber. As fiber from cereals is much less able to stimulate rumen rumination and salivary secretion than that from roughage. It is therefore essential to ensure adequate levels of peNDF in HCDs in order to prevent rumen bloat.

## Conclusions

Feedlot bloat is the most common digestive disorder in fattening ruminant animals, and the cause is attributed to the production of a large amount of stable foam in the rumen when HCDs were fed fattening period. Soluble proteins, soluble polysaccharides and Carboxylates may act as foaming agents and foam stabilizers in the rumen. Supplementation of HCDs with condensed tannin, DSO and other additives may be an effective way to prevent feedlot bloat.

## Author contributions

Conceptualization: YW and LW. Writing—original draft preparation and visualization: YW. Writing—review and editing and supervision: LW and TY. All authors have read and agreed to the published version of the manuscript.
